# S100A4 regulates cell motility and invasion in an *in vitro* model for breast cancer metastasis

**DOI:** 10.1038/sj.bjc.6601483

**Published:** 2004-01-06

**Authors:** S R Jenkinson, R Barraclough, C R West, P S Rudland

**Affiliations:** 1 Molecular Medicine Group, School of Biological Sciences, University of Liverpool, Liverpool L69 7ZB, UK; 2 Department of Public Health, University of Liverpool, PO Box 147, Liverpool L69 3BX, UK

**Keywords:** S100A4, motility, invasion, breast cancer metastasis

## Abstract

Elevated levels of the calcium-binding protein S100A4 are associated with poor patient survival in breast cancer patients and induce metastasis in rodent models. To investigate the effects of S100A4 on different components of the metastatic process, epithelial cells lines have been isolated from nonmalignant tumours in *neu* transgenic mice and from malignant tumours in *neu*/S100A4 double transgenic mice. Additional cell lines expressing both Neu and S100A4 have also been derived by transfection of rat S100A4 cDNA into tumour cell lines cloned from *neu* single transgenic mice. Using these cells in transfilter migration assays, it has been shown that increases in either motility or invasive properties correlate with each other and with the level of S100A4 protein. Injection of three of the cell lines separately into the mammary fat pads of nude mice showed that elevated levels of S100A4 correlated with the degree of metastasis to the lungs. In contrast, changes in cell proliferation and cell–substrate adhesion did not correlate with S100A4 levels. Neither motility nor invasiveness correlated with proteolytic degradation of gelatin as measured by zymography. Thus, the results suggest that the main effect of increases in S100A4 levels in metastasis is to generate increased cell motility and invasion and that this latter change is not dependent upon an increased ability to degrade the intercellular matrix.

The processes by which solid primary tumours, such as those of the breast, are able to disseminate and establish growth at a secondary site are still poorly understood. Tumour metastasis is complex, probably requiring both gain and loss of functions, enabling escape from the primary tumour, and growth at a secondary site. This has led to the search for metastasis-associated genes, which, unlike oncogenes, are unable to initiate tumour formation but are able to induce a metastatic phenotype in previously tumorigenic cells (Davies *et al*, 1993,^1996^; Oates *et al*, 1996). One such metastasis-associated gene is that for the calcium-binding protein S100A4 (Barraclough *et al*, 1987). Increased levels of S100A4 have been shown to induce a metastatic phenotype in several rodent models of mammary carcinogenesis (Davies *et al*, 1993; Grigorian *et al*, 1996). Moreover, elevated expression of S100A4 has been shown to correlate with early patient demise of one group of breast cancer patients (Rudland *et al*, 2000), presumably due to metastatic spread of the primary tumour.

A transgenic murine model that represents human metastatic breast disease in both its pathology and development of metastasis has previously been established by mating MMTV-*neu* transgenic mice with S100A4 transgenic mice (Davies *et al*, 1996). The resultant *neu*/S100A4 transgenic offspring develop both mammary gland tumours and lung metastases, in contrast to the parental transgenic MMTV-*neu* strain, which develops only mammary gland tumours (Bouchard *et al*, 1989; Davies *et al*, 1996), and to the S100A4 transgenic strain, which shows no pathology (Davies *et al*, 1995). Other transgenic murine models showing that overexpression of S100A4 causes metastasis have also been developed (Ambartsumian *et al*, 1996). S100A4 has been shown to be capable of binding to the actomyosin elements of the cellular cytoskeleton (Davies *et al*, 1993; Kriajevska *et al*, 1994; Takenaga *et al*, 1994), raising the possibility that the metastatic effect exerted by S100A4 might occur via its influence on cytoskeletal function, which in turn may regulate cell movement. In this study, new mammary cell lines have been derived from mice carrying transgenes for either the *neu* oncogene alone, or the *neu* oncogene in combination with that for S100A4. The behaviour of these cell lines, and that of the *neu* oncogene containing cell lines transfected with the cDNA for S100A4 *in vitro* have been compared using *in vitro* tests to study individual aspects of S100A4-mediated effects for correlation with *in vivo* phenotypes. In particular, the effect of S100A4 on cytoskeletal-dependent processes, including motility and invasion, as well as on other potential elements of the metastatic phenotype, including metalloproteinase-mediated matrix disruption of an artificial matrix, gelatin, have been examined.

## MATERIALS AND METHODS

### Isolation and characterisation of cell lines

Mixed cell populations were isolated from mammary gland tumour extracts of either *neu* or *neu*/S100A4 transgenic mice (Davies *et al*, 1996), as described previously (Davies *et al*, 1993). Cells isolated from tumour extracts were cloned and subsequently grown in Routine Medium (Dulbecco's modified Eagle's medium (DMEM), 10% (v v^−1^) foetal calf serum (FCS), 100 ng ml^−1^ insulin, 50 ng ml^−1^ hydrocortisone). The cells were tested for the expression of peanut lectin-binding protein and cytokeratin 18 (Jenkinson, 2001), as described previously for rat and human mammary cell lines (Rudland *et al*, 1998). All the cell lines could be grown indefinitely in routine medium and stocks were frozen in liquid nitrogen for preservation. The characterisation and growth of the low S100A4-expressing control cell line Rat mammary (Rama) 25 isolated from a chemically induced rat mammary tumour has been described previously (Bennett *et al*, 1978).

### Transfection of S100A4 DNA into cultured cells isolated from *neu* transgenic mice

For transfection, cell lines grown to approximately 60% confluence in 3 cm diameter dishes were transfected with 0.5 *μ*g of the expression vector for S100A4, pSV2neo-p9Ka (S100A4) (Davies *et al*, 1993) using the Lipofectamine system (Invitrogen Ltd, Paisley, Scotland), following the manufacturer's instructions. At 72 h after transfection, the cells were passaged at a split ratio of 1 : 10 into Selective Medium, Routine Medium containing 0.5 mg ml^−1^ Geneticin (G418). Cells were cultured in this Selective Medium for 2 weeks, the medium being replaced every 2–3 days and the resulting colonies for any one cell line pooled and expanded. Incubation of control nontransfected cells with the Selective Medium caused death of all the cells within 5 days.

### Detection of Neu and S100A4 protein

For Western blotting, total cellular protein was isolated from cell lines; washed twice with cold phosphate-buffered saline (PBS), lysed in 100 *μ*l of 0.125 M Tris-HCl (pH 6.8), 4% (w v^−1^) sodium dodecyl sulphate (SDS), 20% (v v^−1^) glycerol; scraped with a rubber policeman, collected in 1.5 ml tubes and protein quantitated using Bradford's reagent (Pierce and Warriner, Chester, UK) following the manufacturer's instructions. Samples (10 *μ*g) of protein from each cell line in gel loading buffer (0.125 M Tris-HCl (pH 6.8), 4% (w v^−1^) SDS, 20% (v v^−1^) glycerol, 0.04% (w v^−1^) bromophenol blue, 10% (v v^−1^) 2-mercaptoethanol), heated to 100°C, were separated by electrophoresis on 10 or 15% (w v^−1^) polyacrylamide gels in the presence of SDS. The separated polypeptides were transferred to nitrocellulose membranes and incubated with a primary antibody to either c-erbB-2 (Dako Ltd, Ely, Cambs, UK) or S100A4 (Gibbs *et al*, 1995), which were detected, in turn, by means of a peroxidase-conjugated secondary polyclonal antibody and visualised with the Supersignal Chemi-luminescent System (Pierce and Warriner, Chester, UK). Western blotting was performed with protein isolated from three independent experiments. Bands were detected by autoradiography using Fuji X-Omat autoradiography film and quantified by scanning the images using a Shimadzu CS9000 scanning densitometer. Differences in loading were calculated by scanning and quantifying the staining of each of the cell lines on the Coomassie blue stained protein gel.

### Cell substrate-adhesion assay

Cells were trypsinised and counted using a Coulter counter, and resuspended at 2 × 10^5^ cells ml^−1^. Cell suspension (1 ml) was added to each well of a 24-well plate and at time periods of 0.5, 1, 1.5, 2.0, 2.5 h, the number of adherent cells was found by washing the wells three times with PBS to remove any cells in suspension. Any cells adhering to the well were trypsinised and counted using the Coulter counter. Each experiment was carried out in triplicate.

### Cell proliferation assay

Cells were trypsinised and counted as described previously, and resuspended at 1 × 10^4^ cells ml^−1^ and 1 ml was added to each well of a 24-well plate. The cells were incubated for 24, 48, 72 h periods, and at the end of each time period the number of cells was found by trypsinising the cells and counting them in a Coulter counter. Each experiment was carried out in triplicate.

### Motility and invasion assays

The motile and invasive abilities of the cell lines through a filter containing 8 *μ*m pores were measured using a Boyden chamber in a 24-well plate assay system (Corning Costar, High Wycombe, Bucks, UK). Chemotactic-induced motility in response to a FCS concentration gradient was measured by adding 400 *μ*l of Routine Medium containing 10% (v v^−1^) FCS to the lower compartment, and 2 × 10^5^ cells in 200 *μ*l of Routine Medium, but with 2% (v v^−1^) FCS to the upper compartment of the Boyden chamber. The cells were incubated for 24 h, the upper side of the filter was wiped with a cotton swab to remove any nonmotile cells, and the motile cells on the lower side of the filter were fixed and stained using the Diffquik histochemical stain (Dade Behring, Düdingen, Switzerland) according to the manufacturer's instructions. The lower compartment of the Boyden chamber was checked for cells and the number of stained cells/field of 0.50 mm^2^ on the lower side of the filter was counted using a Dynascope with a × 20 objective (Vision Engineering, Woking, Surrey, UK). For each cell line, four experiments were carried out, each experiment consisting of three filters and 10 fields per filter were counted. In controls, fixed numbers of cells incubated for different times up to 24 h showed a linear increase in the number of motile cells on the lower side of the filter. Thereafter, there was an appreciable number of cells present in the lower chamber.

For cell invasion, the filters were coated with 50 *μ*g Matrigel (Collaborative Biomedical Products, Becton Dickinson, Oxford, UK) and the experiments were carried out as for the chemotactic motility assays. The number of invasive cells/field on the lower side of the filters were determined as for the motility assays for three experiments for each cell line.

### Zymography

Cell lines were washed twice with PBS and incubated in 8 ml of growth medium consisting of phenol-red-free DMEM, 2% (v v^−1^) FCS, 200 ng ml^−1^ insulin and 50 ng ml^−1^ hydrocortisone for 48 h. The media were harvested from each of the cell lines, grown to either 40–50% confluency (sparse cultures) or 80–90% confluency (dense cultures) and any protein concentrated into a 1 ml volume or less using an Ultrafree-15 centrifugal filter unit (Biomax-5 K, Millipore, Watford, UK). The concentration of protein in each sample was then determined by measuring the absorbance at 595 nm using Bradford's reagent (Pierce and Warriner, Chester, UK) following the manufacturer's instructions and relating it to the absorbance found for standards containing a known concentration of bovine serum albumin.

Conditioned medium was analysed on 10% (w v^−1^) SDS polyacrylamide resolving gels containing 1 mg ml^−1^ of gelatin, with 4% (w v^−1^) polyacrylamide stacking gels using the Bio-Rad Miniprotean system, as described previously (Heussen and Dowdle, 1980). Briefly, molecular weight markers or samples consisting of 2 or 20 *μ*g of conditioned medium in loading buffer (0.4 M Tris-HCl (pH 6.8), 5% (w v^−1^) SDS, 20% (w v^−1^) glycerol, 0.03% (w v^−1^) bromophenol blue) were loaded onto the gel, without being heated and in the absence of 2-mercaptoethanol. Gels were run at 4°C until the dye front reached the anode end of the gel. The resolving gel was washed for 2 × 30 min in 2.5% (v v^−1^) Triton X-100 to remove the SDS from the gel. Following which the gels were incubated for 18 h at 37°C in substrate buffer (50 mM Tris-HCl (pH 7.5), 10 mM CaCl_2_, 75 mM NaCl). The gel was subsequently incubated in Coomassie blue stain for 1 h and then incubated in destain (45% (v v^−1^) methanol, 5% (v v^−1^) glacial acetic acid) for 1 h, until the digested bands in the gel became clear. The gels were dried on gel drying frames between cellophane sheets (Web Scientific, Crewe, Cheshire, UK).

### Tumorigenicity studies and assays for metastasis

The 8Neu, 8Neup9Ka or PN2 cell lines were injected at 1 × 10^6^ cells in 0.1 ml PBS into three groups of 20, 6-week-old female nude, nu-nu mice, at a single subcutaneous site in the left inguinal mammary fat pad. Mice were autopsied when tumours reached approximately 10% of their body weight, and the tumours, mammary glands, lymph nodes, lungs and any suspicious looking tissues were fixed in Methacarn, processed and embedded in paraffin wax as for the rat (Dunnington *et al*, 1983). Samples were sectioned and stained with haematoxylin and eosin. Further sections were immunocytochemically stained with antibodies to either c-erbB-2 (Dako Ltd, Ely, Cambs, UK) or S100A4 (Gibbs *et al*, 1995), using a peroxidase-conjugated secondary antibody and photographed using a Reichert Polyvar microscope, as described previously (Davies *et al*, 1996). Sections of both lungs and lymph nodes for each animal were examined for metastases. Animals were maintained under UK Home Office Project Licence no. 40/1515 to Professor PS Rudland, in accordance with the guidelines set down by the United Kingdom Coordinating Committee for Cancer Research (Workman *et al*, 1998).

## RESULTS

### Establishment of clonal cell lines

Cells were initially isolated from frozen samples of mammary gland tumours from both *neu* and *neu*/S100A4 transgenic mice (Materials and Methods). The tumours were selected from mice representative of the general phenotype of the transgene status, as demonstrated by haematoxylin and eosin-stained tumour sections. Three permanently growing cell clones were established from the mammary gland tumours of a *neu* transgenic mouse and termed 4Neu, 5Neu and 8Neu. Two permanently growing cell clones were established from the mammary gland tumours of a bitransgenic *neu*/S100A4 mouse and termed PN1 and PN2. The stable transfection of the 4Neu and 8Neu clonal cell lines isolated from the *neu* transgenic mouse with the rat pSV2neo-p9Ka (S100A4) expression vector yielded two further cell lines obtained from the pooled transfectant clones, termed 4Neup9Ka and 8Neup9Ka, respectively, which grew in Geneticin-containing medium. Parallel mock transfections without the expression vector yielded no colonies after incubation of cells in Geneticin-containing medium. All of the isolated cell lines were epithelial in nature as determined by staining for epithelial lineage markers such as Cytokeratin 18 and peanut lectin-binding protein (not shown) (Jenkinson, 2001).

Each of the cell lines had a different expression profile for S100A4 and Neu proteins, as determined by Western blotting (Table 1

**Table 1:** Expression levels of Neu, S100A4 proteins and invasive and motile behaviour of cell lines

**Cell line^a^**	**Neu protein^b^ (arbitrary units)**	**S100A4 protein^c^ (arbitrary units)**	**Adhesion^d^ (%)**	**Growth rate^e^ (cells h^−1^)**	**Motility^f^ (cells per field)**	**Invasion^g^ (cells per field)**
5Neu	2.83±0.42	1.13±0.37	50.6±3.4	546±18	N/D	N/D
4Neu	3.24±0.34	1.59±0.37	30.8±2.8	2111±13	40.8±14.4	8.08±3.52
4Neup9Ka	2.75±0.45	3.12±0.31^h^	43.5±5.3	1058±47	129±19.8^h^	25.8±2.21^h^
8Neu	3.64±0.70	0.09±0.04	24.2±1.6	1104±34	6.95±3.71	3.21±0.68
8Neup9Ka	5.55±0.85	1.13±0.18^h^	32.9±5.2	2830±183	29.5±6.6^h^	10.7±1.92^h^
PN1	5.80±1.19	1.66±0.18	56.1±1.3	2096±49	75.0±21.6	13.2±6.43
PN2	4.20±0.54	1.88±0.09	48.2±0.8	1730±88	111±46.0	23.8±1.98^i^

a Cell lines were isolated as described in Materials and Methods.

b Relative protein values for Neu and S100A4 are normalised to the low Neu-, low S100A4-expressing cell line Rama 25, after correcting for differences in the Coomassie blue-stained protein gel, mean±s.e. of three experiments (Materials and Methods).

c Relative protein values for Neu and S100A4 are normalised to the low Neu-, low S100A4-expressing cell line Rama 25, after correcting for differences in the Coomassie blue-stained protein gel, mean±s.e. of three experiments (Materials and Methods).

d Cell substrate-adhesion was measured at 0.5 h and is expressed as a mean percentage of cells that had adhered±s.e. of three experiments for 2 × 10^5^ cells plated.

e Cell growth rate in cells per hour is the mean±s.e. of three experiments for 1 × 10^4^ cells plated.

f Cell motility, average cells/field for 4 experiments±s.e. (Materials and Methods). Each experiment consisted of three filters per cell line and 10 fields per filter were counted.

g Cell invasion through Matrigel, average cells per field for three experiments±s.e. (Materials and Methods). Each experiment consisted of three filters per cell line and 10 fields per filter were counted.

h Significantly higher for the S100A4-transfected cell line compared to its parental cell line (Student's *t*-test, *P*⩽0.03).

i Significantly higher for the cell lines isolated from the double transgenic *neu*/S100A4 mice than the single transgenic *neu* mice (Student's *t*-test, *P*⩽0.02). ND=not determined, since this cell line had no S100A4-transfected derivative with which to compare motile and invasive abilities.

, Figure 1

**Figure 1:**
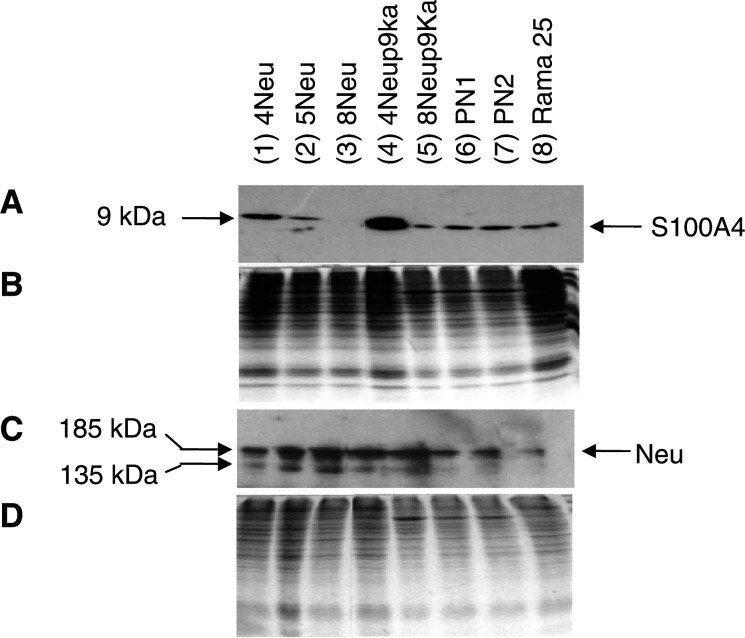
Detection of S100A4 and Neu protein in cell lines isolated from mammary tumours of transgenic mice. Cellular protein extracts were analysed by Western blotting for (**A**) S100A4 and (**C**) Neu protein on SDS–polyacrylamide gels. Lanes 1, 2 and 3 contain 10 *μ*g of protein from the cell lines isolated independently from a *neu* transgenic mammary gland tumour: 4Neu, 5Neu and 8Neu. Lanes 4 and 5 contain 10 *μ*g of protein from S100A4-transfected, *neu*-transgenic-derived cell lines: 4Neup9Ka and 8Neup9Ka. Lanes 6 and 7 contain 10 *μ*g of protein from cell lines isolated from a *neu*/S100A4 bitransgenic mammary gland tumour: PN1 and PN2. Lane 8 contains 10 *μ*g of protein from a low S100A4, low Neu-expressing rat cell line: Rama 25. Coomassie blue staining of the protein on the SDS–polyacrylamide gels is shown for the same gels as those immunocytochemically stained for (**B**) S100A4 and for (**D**) c-erbB-2.

). The S100A4 content of the mouse cell lines varied from about a tenth to over three times that of the known low S100A4-expressing control Rama 25 cell line isolated from a dimethyl benzanthracene-induced rat mammary tumour. Cell lines transfected with the S100A4 cDNA, 4Neup9Ka and 8Neup9Ka showed a two to 12-fold increase in S100A4 compared to their parental 4Neu and 8Neu cell lines (Student's *t*-test, *P*<0.05). The correct sized protein of apparent molecular weight 9 kDa was seen for all cell lines (Figure 1A). The Neu protein was expressed in all of the mouse cell lines, there being about a two-fold difference between the highest PN1 and lowest 4Neup9Ka cell lines. The control Rama 25 cell line showed a level of endogenous Neu gene expression significantly lower than all of the mouse cell lines including 4Neup9Ka (Student's *t*-test, *P*<0.05). The correct sized protein of 185 kDa was seen for all the cell lines, but an additional band at 135 kDa was sometimes seen; this may have been due to crossreactivity with the EGF receptor (Figure 1C). The *neu* transgene retained its activating transmembrane point mutation at nucleotide position 2012, as determined by sequencing of PCR-amplified mRNA isolated from the cell lines (Jenkinson, 2001).

### Comparison of cell substrate-adhesion and proliferation with S100A4 levels

**Figure 2:**
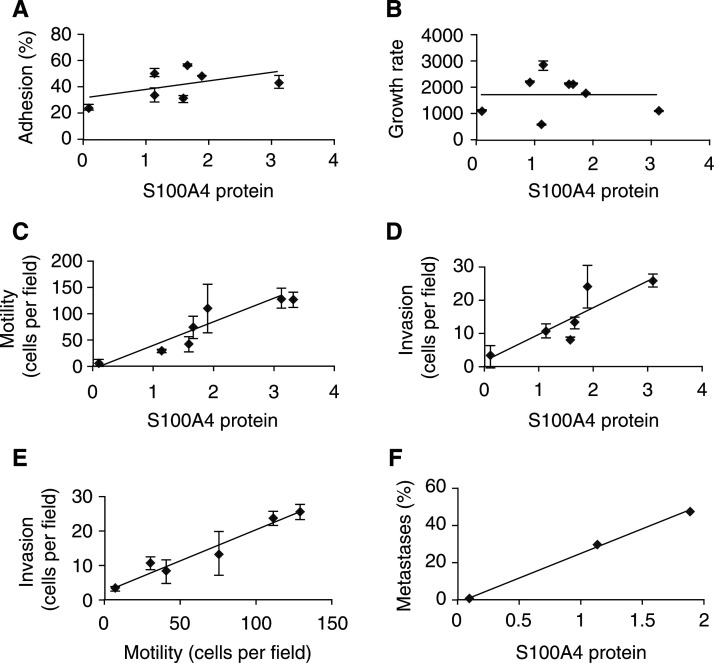
Relationship between the levels of S100A4 protein, growth rate, substrate adhesion, cell motility, invasion and metastatic potential of the cell lines. (**A**) substrate adhesion (adhesion); (**B**) growth rate; (**C**) motility and (**D**) invasion of each cell line are plotted against the relative levels of S100A4 protein. (**E**) Motility of each cell line is also plotted against its invasion. The data for the cell lines (4Neu, 5Neu, 8Neu, 4Neup9Ka, 8Neup9Ka, PN1, PN2) are taken from Table 1. (**F**) Relative levels of S100A4 protein are plotted against the percentage of tumour-bearing mice with lung metastases for each of the cell lines: 8Neu, 8Neup9Ka and PN2 (data taken from Table 2). Least-squares regression analysis of a fit of the points to a straight line yields for (**A**) regression coefficient *R*^2^=0.23, probability *P*=0.27; (**B**) R^2^=0.0002, *P*=0.97; (**C**) *R*^2^=0.822, *P*=0.012; (**D**) *R*^2^=0.765, *P*=0.0022; (**E**) *R*^2^=0.950, *P*=0.00096; and (**F**) *R*^2^=0.996, *P*=0.038.

Preliminary experiments showed that 0.5 h after plating, 25–50% of the total cells in the assay had adhered to the plastic substratum and therefore 0.5 h was taken as an appropriate time period over which to measure rate of adhesion. The substrate adhesion of the different cell lines varied. The percentage of total cells adhering at 0.5 h was plotted against the levels of S100A4 protein (Figure 2A

), as determined by Western blotting, for each cell line. Cell adhesion to plastic was found not to correlate significantly by least-squares regression analysis with S100A4 protein levels (correlation coefficient *R*^2^=0.23; probability points lie on a straight line, *P*=0.27) (Figure 2A), nor with Neu (*R*^2^=0.036; *P*=0.71) protein levels (Table 1).

The rate of cell proliferation measured during the exponential phase of growth varied for each cell line. To examine whether cell proliferation was associated with S100A4 expression, the rate of cell proliferation was plotted against the protein levels of S100A4 as determined by Western blotting for each cell line (Figure 2B). Cell proliferation rates did not correlate with levels of S100A4 protein by least-squares regression analysis (*R*^2^=0.002; *P*=0.97) (Figure 2B), although they did with levels of the Neu protein (*R*^2^=0.6; *P*=0.04) (data in Table 1).

### Cell motility *in vitro* and levels of S100A4

Chemotactic motility was measured as described in Materials and Methods. Motility was determined over a 24 h period, a time period, which was shown to ensure that all motile cells remained attached to the lower side of the filter (Jenkinson, 2001). Cell motility varied from about seven to nearly 130 migrating cells per field (Table 1). Motility of the S100A4-transfected cell lines 4Neup9Ka, 8Neup9Ka was significantly higher than their respective parental cell lines 4Neu and 8Neu (Student's *t*-test, *P*<0.03) (Table 1). Moreover, the motilities of cell lines isolated from the *neu*/S100A4 transgenic mice (PN1, PN2) were higher than those isolated from *neu* transgenic mice (4Neu, 8Neu) (Table 1).

Comparison of S100A4 levels with cell motility for the different cell lines showed that S100A4 levels and motility were closely associated (Table 1). This difference in cell motility and S100A4 protein levels is significant when 8Neu is compared individually to all the other cell lines (Student's *t*-test, *P*<0.05). When S100A4 protein levels are plotted against motility, there is a significant linear relationship when assessed by a least-squares regression analysis (*R*^2^=0.822; *P*=0.012) (Figure 2C). In the case of Neu, no correlation was found between cell motility and Neu protein levels for the different cell lines, by least-squares regression analysis (*R*^2^=0.0061; *P*=0.88) (Table 1).

### Cell invasion *in vitro* and levels of S100A4

To allow for direct comparison between cell invasion and cell motility, the invasive abilities of the cell lines were measured using the assay conditions optimised for the motility assay but with a coating of Matrigel on the filter separating the upper and lower compartment of the Boyden chamber. Using this assay, cell invasion for the different cell lines varied from about three to nearly 26 invading cells per field (Table 1). The invasive ability was significantly higher in the S100A4 transfected cell lines 4Neup9Ka, 8Neup9Ka than their corresponding Neu parental cell lines 4Neu, 8Neu (Student's *t*-test, *P*<0.03) (Table 1). Moreover, the invasive abilities of cell lines isolated from the *neu*/S100A4 transgenic mice (PN1, PN2) were higher than those isolated from neu transgenic (4Neu, 8Neu), that for PN2 was significantly so (Student's *t*-test, *P*<0.02).

A close association was also observed between increasing S100A4 protein levels and increasing invasive ability of the cell lines (Table 1). All of the cell lines expressed a significantly higher level of S100A4 protein than the 8Neu cell line (Student's *t*-test, *P*<0.01) and demonstrated a significantly higher invasive ability when compared individually to 8Neu (Student's *t*-test, *P*<0.01). A significant linear correlation between S100A4 protein levels and invasive ability was demonstrated by least-squares regression analysis for each cell line (*R*^2^=0.765; *P*=0.022) (Figure 2D). However, the level of Neu protein was unrelated to the invasive abilities of the cell lines, since increasing levels of Neu protein did not correlate with increasing cell invasion (*R*^2^=0.0006; *P*=0.96) (Table 1).

To examine whether the invasive abilities of the cell lines correlated with those of their motile properties, these two parameters were compared for each cell line. A plot of cell motility against cell invasion for six cell lines approximated to a straight line and showed a strong correlation between these two parameters using least-squares regression analysis (*R*^2^=0.95; *P*=0.00096) (Figure 2E). Interestingly, the 4Neu cell line and its S100A4-transfected derivative cell line 4Neup9Ka showed the same five-fold difference between motility and invasion, and the 8Neu cell line and its derivative transfectant 8Neup9Ka showed a similar difference between motility and invasion of 2.2- and 2.7-fold, respectively (Table 1). It would therefore seem that the invasive abilities of the cell lines through Matrigel are directly related to their motile abilities *in vitro*.

### Matrix metalloproteinase production and levels of S100A4

Matrix metalloproteinase (MMP) activities were measured by gelatin zymography of conditioned media harvested from cell lines grown in Routine Medium supplemented with 2% (v v^−1^) FCS, the same medium as that used in the upper chamber of the Boyden system during the invasion assay. To analyse any effect of cell confluency on MMP production, medium was harvested from sparse cultures of cells, grown to 40–50% confluency and from dense cultures of cells grown to 80–90% confluency.

**Figure 3:**
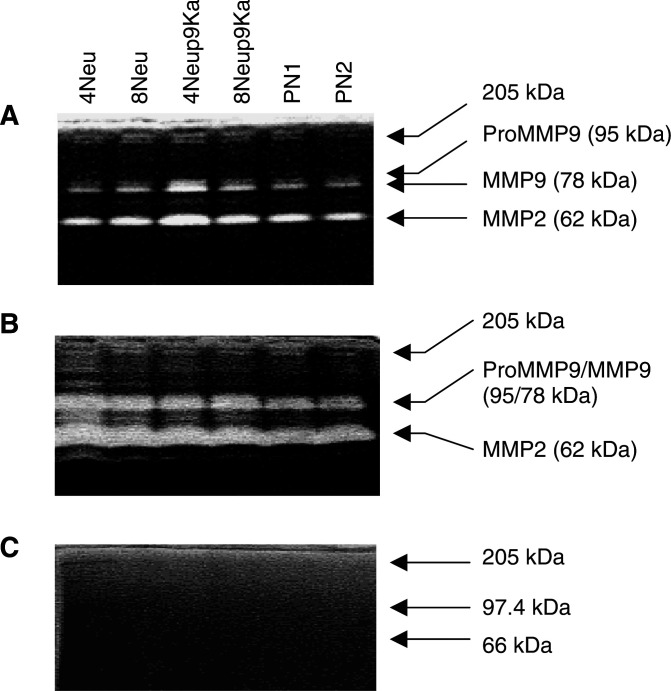
Zymogram analysis of matrix metalloproteinase production by cell lines. Conditioned medium harvested from each cell line plated at a low density for (**A**) 2 *μ*g protein, (**B**) 20 *μ*g protein, and at a high density for (**C**) 20 *μ*g protein was analysed by gelatin zymography for the presence of both pro and active MMPs, as described in Materials and Methods.

Bands of lysed gelatin were detected in 2 and 20 *μ*g samples of the conditioned medium of sparsely grown cultures (Figure 3A, B

), but no bands were present in the same amounts of conditioned medium isolated from dense cell cultures (Figure 3C). Analysis of 2 *μ*g of protein from conditioned medium showed clear bands at a size corresponding to proMMP9 (*M*_r_ 92 kDa) and MMP9 (*M*_r_ 78 kDa) (Figure 3A). A single band was seen at 67 kDa, which corresponds to the size of MMP2 (Figure 3A). When 20 *μ*g of protein from conditioned medium was analysed, the separation between proMMP9 and MMP9 bands was lost and a large band of lysis was also seen at a size corresponding to MMP2 (Figure 3B). In sparse cultures, the amount of digestion was dependent on the amount of total protein (compare Figure 3A and B). The molecular mass of the lysed bands and the intensity of the bands of digestion of the gelatin substrate was, however, the same for each cell line for either 2 or 20 *μ*g of protein from conditioned medium. This result provided semiquantitative evidence that each of the cell lines from these sparse cultures produced approximately the same amount of both pro and active MMPs. In contrast, conditioned medium from the dense cultures contained no detectable pro or active MMPs (Figure 3C). No attempt was made to measure MMP activity in cells or cell membranes.

### Incidence, metastatic ability and pathology of tumours produced by selected cell lines

In all, one million cells from the low S100A4-expressing 8Neu cell line, from the moderate S100A4 expressing 8Neup9Ka and from the high S100A4-expressing PN2 cell line were injected into the mammary fat pads of female nude mice. All mice produced mammary gland tumours after a period of 2–3 weeks (100% incidence) (Table 2

**Table 2:** Incidence of mammary gland tumours and lung metastases in nude mice injected with cell lines

**Cell line injected^a^**	**Total number of mice^b^**	**Number of mice with mammary gland tumours^c^ (%)**	**Number of mice with metastases^d^ (%)**
8Neu	19	19 (100%)	0 (0%)
8Neup9Ka	20	20 (100%	6^e^,^f^ (30%)
PN2	19	19 (100%)	9^f^,^g^ (48%)

a Cell lines representative of three different cell types, those isolated from *neu* transgenic mice (8Neu), their S100A4-transfected derivatives (8Neup9Ka) and those isolated from *neu/S*100A4 double transgenic mice (PN2) were injected into the mammary fat pad of female nude mice.

b Total number of mice injected for each cell line.

c Number of mice with palpable mammary gland tumours 3 weeks after inoculation with cell lines; percentage of mice bearing tumours (%) is also shown.

d Number of mice with metastases in the lungs; percentage of tumour-bearing mice with metastases (%) is also shown.

e All six of the lung metastases were classified as micrometastases, <0.1 mm diameter.

f Significantly higher number of metastases than the 8Neu cell line (Fisher's exact test; 8Neup9Ka, *P*=0.028; PN2, *P*=0.005).

g Two of the lung metastases were classified as macrometastases >1 mm diameter, and the remaining seven lung metastases were classified as micrometastases <0.1 mm diameter.

).

**Figure 4:**
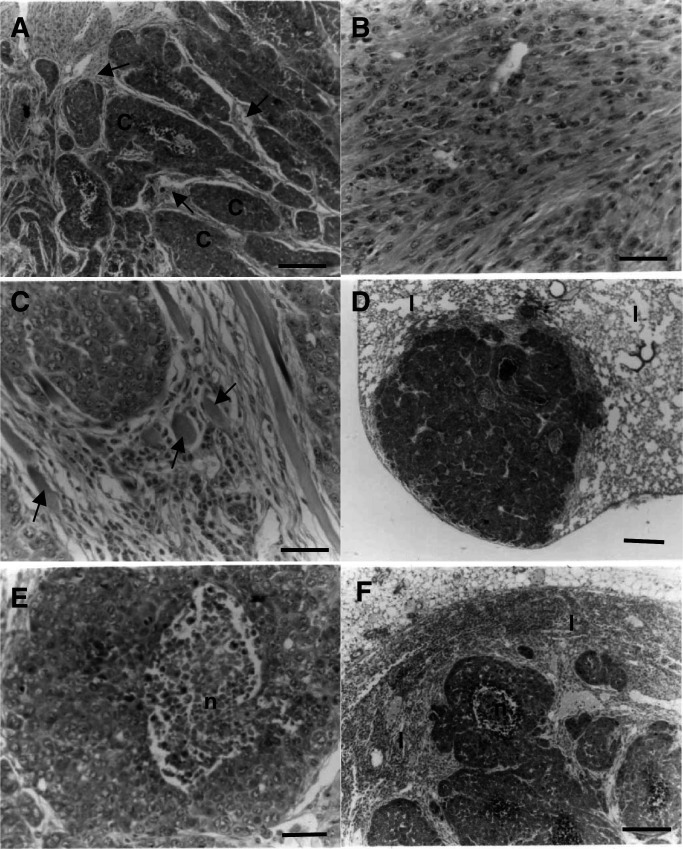
Histopathological appearance of cell lines growing as tumours in nude mice. (**A**) Mammary tumour produced by PN2, showing chord-like structures *(c)*, surrounded by spindle cells (arrows). (**B**) Mammary tumour produced by PN2, showing a predominant spindle cell component. (**C**) Mammary tumour produced by 8Neup9Ka, showing invasion of muscle (arrows). (**D**) Lung *(l)* macrometastasis produced by a PN2-derived tumour, showing chord-like structures. (**E**) Higher magnification of PN2-derived lung macrometastasis in (**D**) above, showing chord-like structures with central necrosis *(n)*. (**F**) Lymph node *(l)* metastasis produced by an 8Neup9Ka-derived tumour, showing chord-like structures with central necrosis *(n)*. Magnification, (**A**, **B**, **C**) × 220; (**D**) × 22; (**E**) × 175; (**F**) × 55. *Bars*, (**A**, **B**, **C**, **E**) 50 *μ*m; (**D**, **F**) 200 *μ*m.

The primary tumours consisted of chord-like structures (Figure 4A

) with varying amounts of spindle cells (Figure 4B), but only the 8Neup9Ka and PN2 cell lines showed local invasion of the muscle (Figure 4C) and produced lung metastases (Figure 4D) (Table 2). For the moderate S100A4-expressing 8Neup9Ka cell line, all of the lesions in the lung were classified as micrometastases, less than 0.1 mm in diameter. For the PN2 cell line, two mice produced macrometastases, greater than 1 mm in diameter, and the remaining seven mice, which produced lesions in the lung, were classified as micrometastases, as above (Table 2). The histology of the lung metastases was similar to the primary with chords of cells (Figure 4E), often with central necrosis for the larger lesions (Figure 4E). Both the moderate S100A4-expressing 8Neup9Ka cell line and the high S100A4-expressing PN2 cell line produced a statistically significant greater number of lung metastases than the low S100A4-expressing 8Neu cell line (Fisher's exact test, *P*<0.03). The incidence of metastasis in the high S100A4-expressing PN2 cell line was also greater than that in the moderate S100A4-expressing 8Neup9Ka cell line, although not statistically significant (Fisher's exact test, *P*=0.18). Furthermore, when the level of S100A4 protein in the cultured cells was plotted against the percentage of tumour-bearing mice with lung metastases, a straight line could be drawn through the three points (least-squares regression analysis *R*^2^=0.9965; *P*=0.038) (Figure 2F). In contrast, no such linear relationship occurred between the levels of Neu protein and the percentage of mice with metastases (*R*^2^=0.18; *P*=0.72; graph not shown). Metastases were also observed in the inguinal lymph nodes of some of the mice injected with the 8Neup9Ka and PN2 cell lines; however, these were not included in any statistical analyses, since lymph node tissue was not available from all of the injected mice.

**Figure 5:**
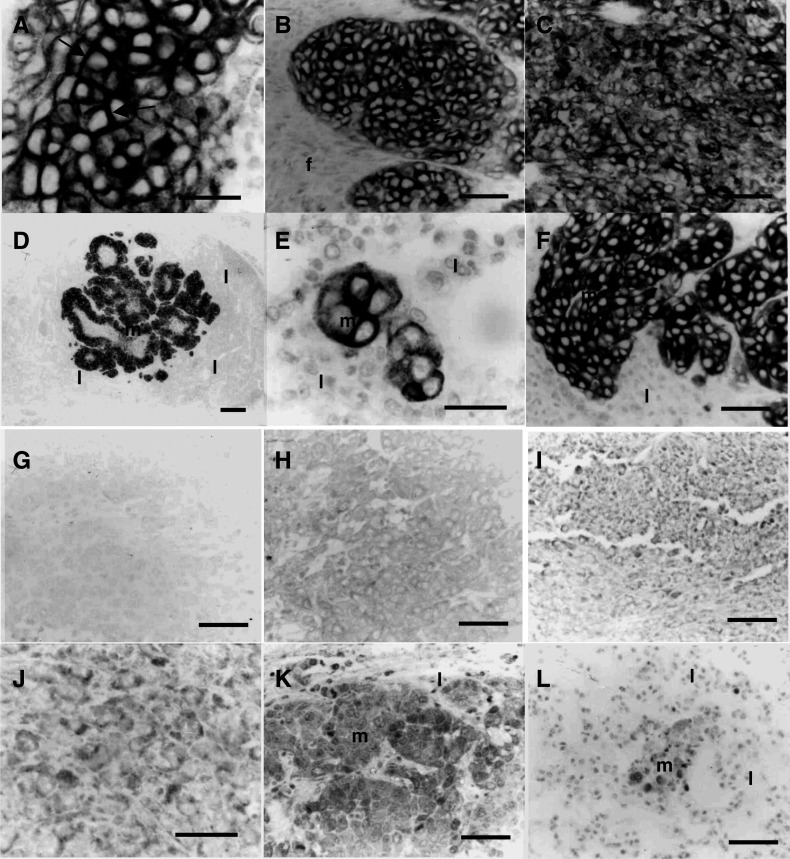
Immunocytochemical staining of mammary tumours and metastases produced in nude mice. (**A**) Mammary tumour produced by 8Neu, showing strong cell surface staining of carcinoma cells for Neu (arrows). (**B**) Mammary tumour produced by PN2, showing strong staining of chords of epithelial cells for Neu *(e)*, the stromal fibroblasts *(f)* were unstained. (**C**) Mammary tumour produced by PN2, showing strong staining of spindle cells for Neu. (**D**) Lymph node metastasis produced by an 8Neup9Ka-derived tumour, showing strong staining for Neu *(m)*, but no staining of lymphoid cells *(l)*. (**E**) Lung micrometastasis produced by an 8Neup9Ka-derived tumour, showing strong staining for Neu *(m)*, but no staining of parenchymal lung tissue *(l)*. (**F**) Lung metastasis produced by a PN2-derived tumour, showing strong staining for Neu *(m)*, but no staining of lung parenchymal tissue *(l)*. (**G**) Mammary tumour produced by 8Neu, showing no staining above background for S100A4. (**H**) Mammary tumour produced by 8Neup9Ka showing low/moderate staining for S100A4. (**I**) Mammary tumour produced by PN2, showing moderate staining of spindle cells for S100A4. (**J**) Higher magnification of a mammary tumour produced by PN2 showing moderate/strong cytoplasmic staining for S100A4. (**K**) Lymph node metastasis produced by an 8Neup9Ka-derived tumour, showing strong staining for S100A4 *(m)*, but no staining of lymphocytes *(l)*. (**L**) Lung micrometastasis, showing strong staining for S100A4 *(m)*, but no staining of lung parenchymal tissue *(l)*. Magnification, (**A**, **E**, **J**) × 550; (**B**, **C**, **F**, **G**, **H**, **I**, **K**, **L**) × 220; (**D**) × 22. Bars, 50 *μ*m; (**A**, **E**, **J**) 25 *μ*m; 50 *μ*m; (**B**, **C**, **F**, **G**, **H**, **I**, **K**, **L**); (**D**) 200 *μ*m.

Immunocytochemical staining for Neu showed that all three cell lines 8Neu (Figure 5A

), 8Neup9Ka and PN2 (Figure 5B) produced strongly-staining primary tumours, even when they were predominantly composed of spindle cells (Figure 5C). Lymph node metastases (Figure 5D), the micrometastases (Figure 5E) and the macrometastases (Figure 5F) in the lungs were also stained strongly for Neu. In contrast, immunocytochemical staining of the same lesions for S100A4 depended on the identity of the cell line injected into the nude mice. Thus, no staining for S100A4 was seen in the tumours produced by the 8Neu cell line (Figure 5G). The tumours produced by the 8Neup9Ka cell line showed a low to moderate staining for S100A4 (Figure 5H), which varied within each tumour. Tumours produced by the PN2 cell line showed higher levels of staining for S100A4 than the tumours produced by the other two cell lines, particularly for the spindle cell component (Figure 5I, J). Immunocytochemical staining of the macrometastases for S100A4 usually showed a stronger intensity of staining than was present in the primary tumours (compare Figures 5K and I), and all the micrometastases in the lungs were stained strongly (Figure 5L). There was no immunocytochemical staining of lymphocytes observed in the lymph nodes of the nude mice (Figure 5K). Although no immunocytochemical staining for Neu or S100A4 was found in the lung tissue of the nude mice bearing mammary gland tumours produced by the 8Neu cell line (not shown), clusters of stained cells forming micrometastases and even single cells were readily discernable in the lungs of mice with tumours produced by the 8Neup9Ka and PN2 cell lines (Figure 5E, L). These results confirmed that the tumour cells observed in the lungs were derived from the injected cell lines and not from the parenchymal lung tissue, and that these cells had arisen from the primary tumours and metastasised to the lungs.

## DISCUSSION

Cell lines expressing elevated levels of S100A4 have been obtained either by direct transfection of cell lines from mice carrying the *neu* transgene or by isolation of cell lines from mice carrying both the *neu* and the S100A4 transgenes. The former mice produce only nonmetastatic tumours, while the latter mice produce metastatic tumours (Davies *et al*, 1996). The establishment of these cell lines expressing different levels of Neu and S100A4 has provided the opportunity to examine the effects of different levels of these proteins on cell behaviour *in vitro*, which may be related to metastasis *in vivo*. There was no correlation between the levels of S100A4 and cell-substrate adhesion to plastic substrata or proliferation rates for this particular set of cell lines; cell–cell adhesion was not measured. These data are the first to demonstrate in a transgenic murine-derived tumour system that S100A4 levels correlate positively in a linear fashion with cell invasive ability through Matrigel in response to an FCS density gradient. This result demonstrates also that S100A4 causes, either directly or indirectly, the enhanced invasive ability in at least the S100A4-transfected cell lines. In contrast, correlation of levels of Neu protein with only proliferation rates and not with substrate adhesion, motility or invasion is consistent with the expression of the *neu* transgene causing the largely proliferative breast lesions and noninvasive breast tumours observed in transgenic mice (Davies *et al*, 1996) and rats (Davies *et al*, 1999). To exclude the possibility that any correlations were obtained by chance with the cell lines reported here (Table 1), the analyses were expanded to include a further two cell lines with differing levels of Neu and S100A4 from another *neu* transgenic mouse tumour. These analyses still yielded the same significant correlations as before (Jenkinson, 2001).

Whether production of S100A4 correlates with invasive ability has remained equivocal. Previously, S100A4 had been shown to correlate with the invasive ability *in vitro* of Lewis lung carcinoma cell lines (Takenaga *et al*, 1994). However, in a previous murine mammary gland-derived cell line system, S100A4 levels were found not to correlate with invasive ability through Matrigel (Ford *et al*, 1995). In that system, invasive ability was observed for only one S100A4-expressing cell line. Comparison of this invasive cell line to the other S100A4-expressing, noninvasive cell lines in that study, demonstrated that the invasive cell line was the only one to possess proteolytic activity, at least *in vitro* (Ford *et al*, 1995). Zymogram analysis of proteolytic activity in the cell lines isolated here showed that MMP2 and MMP9 were secreted in both pro and active forms at similar levels, in all of the cell lines, independently of levels of S100A4. Thus, in our present system, S100A4 would appear not to regulate cell invasion by increasing proteolytic activity either in low- or high-density cultures. Since MMP activity in membranous fractions was not measured, we cannot exclude the possibility that differences in these activities occurred in our systems and that these differences could be regulated by S100A4.

Matrix metalloproteinase production could, however, be regulated in all of the cell lines by confluency in culture. Matrix metalloproteinase activity was observed only in the conditioned medium of sparsely plated cells. It would therefore seem that complete cell–cell contact provides an inhibitory extracellular signal, which prevents MMP production/secretion in these cells. Previous studies have also demonstrated that proteolytic activity is dependent on cell density. For example, in the media of sparse astrocytoma cells, more collagenase activity was seen than in dense cultures (Tamaki *et al*, 1996), and in epidermoid carcinoma cells, MMP9 secretion was detected only in sparse cultures (Xie *et al*, 1994). Furthermore, MMP2 binding to the cell surface of breast carcinoma cells was detected only in low-density cultures (Menashi *et al*, 1998). The most likely explanation for this observation would be self-preservation of the tumour, as MMP production in a tight mass of tumour cells would possibly lead to tumour cell degradation. The findings reported here are, therefore, in accordance with those of the Lewis lung carcinoma cell line model described above, where MMP9 was produced in all of the cell lines, independently of the level of S100A4 (Takenaga *et al*, 1994), but contrast with studies in an osteosarcoma cell system which implicated S100A4 in the control of both the levels of MMPs and of the tissue inhibitors of matrix metalloproteinases (TIMPs) (Bjornland *et al*, 1999). In the latter system, the reduction in S100A4 expression in these cells resulted in downregulation of mRNA levels for MMP2, MT-MMP1 and TIMP-1, and upregulation of TIMP-2. The levels of TIMPs produced in the cell system isolated and characterised in this study have not been examined. Thus, it is still possible that TIMPs may be differentially expressed in our cell lines and thereby inhibit MMP activity to a different extent in each of the cell lines, although their role *in vivo* is more complex (Hayakawa *et al*, 1992; Sato *et al*, 1994; Ree *et al*, 1997; Yoshiji *et al*, 1998; Remacle *et al*, 2000).

In contrast to gelatinase activity, motility, a second major component of invasion examined *in vitro*, showed a strong linear correlation with levels of S100A4 protein in the different cell lines. The stimulus for motility did not need to be directional, since chemotactic and chemokinetic stimuli provided by 10% FCS, induced the same degree of motility (Jenkinson, 2001). However, 2% FCS was only able to stimulate minimal motility of the cell lines (Jenkinson, 2001), suggesting that a threshold level of motility-stimulating molecules are probably required for cell motility. These results *in vitro* suggest that an appropriate and sufficient chemical stimulus is probably required for cell migration *in vivo*. The results demonstrated here are in accordance with previous findings in other cell systems that increased S100A4 levels results in increased cell motility (Takenaga *et al*, 1994; Ford *et al*, 1995). A significant linear correlation between S100A4 and cell motility, however, has only been previously demonstrated in the Lewis lung carcinoma cell system by measuring the phagocytic tracks of cells in the absence of a chemotactic stimuli (Takenaga *et al*, 1994). The results presented in this study are, therefore, the first to demonstrate in a mammary tumour cell system, that cell motility correlates with levels of S100A4 in a statistically significant linear fashion and that, at least in the transfected cell lines, S100A4 is the cause of this change. Furthermore, cell motility was shown to correlate strongly with cell invasion. The fact that the gradient of the plot of S100A4 against motility is about five times that of the plot of S100A4 against invasion (Figure 2C, D) suggests that the same cells are having to penetrate a more resistant barrier. However, since both plots are linear and the plot of invasion against motility is also linear with a similar gradient (Figure 4E), the three parameters are likely to be closely related. It is therefore probable that S100A4-induced increases in invasive ability are a result of S100A4-driven cell motility in the cell lines under study. The requirement for proteolytic activity for successful cell invasion should not, however, be underestimated, since previous reports have shown that high S100A4-expressing mammary tumour cell lines, which were shown to be motile *in vitro*, were not invasive *in vitro* or *in vivo*, due to a lack of MMP production (Ford *et al*, 1995). This difference may reflect differences between the two mouse mammary systems under study. The transgenic mouse system reported here may produce sufficient levels of MMPs for motility to be rate limiting for invasion, whereas for the mouse system of Ford *et al* (1995) this is not the case.

The mechanisms whereby S100A4 modulates cell motility are unknown. However, S100A4 is known to bind to various components of the actin–myosin cytoskeleton (Watanabe *et al*, 1993; Kriajevska *et al*, 1994; Takenaga *et al*, 1994). In addition, recent advances in DNA microarray technology have led to the isolation of several other metastasis-associated genes, many of the products of which are associated with the cellular cytoskeleton (Khanna *et al*, 2001) and its regulation (Clark *et al*, 2000). The ability of S100A4 to bind and thereby regulate the phosphorylation of nonmuscle myosin heavy chain *in vitro* (Kriajevska *et al*, 2000), *inter alia* (Chen *et al*, 2001), may indicate that S100A4 plays a role in regulating cellular dynamics. A precedent has already been set for myosin phosphorylation as a mechanism of regulating cell motility. The Rho family of small GTPase proteins regulates motility by regulating actin–myosin dynamics via phosphorylation of both the myosin heavy and light chains (Amano *et al*, 1996; Kimura *et al*, 1996). The Rho family has, however, been fairly extensively studied in the context of cell motility, while relatively little is known, as yet, about the effector mechanisms of S100A4 and other metastasis-related proteins in mediating motility.

The tumorigenic and metastatic abilities of the cell lines were examined in the immunoincompetent, nude mouse, without the complication of an immune response. The isolation and culture of tumour cells from both *neu* and *neu*/S100A4 transgenic mice increased the efficiency of mammary tumour production from 44 and 68% as seen after 14 months in *neu* and *neu*/S100A4 transgenic mice (Davies *et al*, 1996), respectively, to 100% in the nude mice for all of the injected cell lines. This increase in efficiency of production of primary tumours was probably due to the following reasons. Firstly, constitutive expression of *neu* was observed in the cell lines. There was no requirement to add hormones *in vitro* or to mate *in vivo* to stimulate the expression of *neu*, whereas in the original transgenic mice the *neu* transgene requires the hormones of pregnancy to activate its MMTV promoter (Bouchard *et al*, 1989; Davies *et al*, 1996). Secondly, in the *neu* transgenic mice all of the epithelial cells express the *neu* transgene, but only some cells are able to form tumours. This latter result indicates that a second genetic change is required, in addition to expression of the activated *neu* transgene, to allow tumour formation. One possible explanation is the high level of the loss of heterozygosity, a fact which may be related to the loss of tumour suppressor genes, which has previously been observed in these *neu* transgenic mice (Cool and Jolicoeur, 1999). Since cell–cell adhesion was not measured, the possible role of E-cadherin as a tumour suppressor in these systems is unknown. Whatever the change required, this event had clearly taken place in the tumours from which the cell lines were isolated.

The frequency of tumour metastasis, however, remained the same in the *neu*/S100A4 tumour-derived PN2 cell line as in the *neu*/S100A4 transgenic mice, with only 50% of mice with mammary gland tumours developing lung metastases. The efficiency of tumour metastasis for the 8Neup9Ka cell line was lower, with only 30% of mice with mammary gland tumours developing lung metastases. The 8Neu cell line developed no lung metastases. These *in vivo* results follow the same pattern observed for the cell lines in *in vitro* assays, where the 8Neu cell line had a low level, the 8Neup9Ka cell line a moderate level, and the PN2 cell line a high level of both motility and invasion. Confirmation that these metastases are derived from the *neu*/S100A4-expressing mammary gland tumours is obtained by positive staining of these metastases for both Neu and S100A4. The level of metastasis expressed as a percentage of afflicted animals was found to be positively correlated with the level of S100A4, but not with the level of Neu protein in the cell lines, albeit for only three of the cell lines. Metastasis in these Neu-expressing cell lines is therefore clearly dependent, either directly or indirectly, on the level of S100A4 protein.

In this transgenic mouse model system, *neu* acts as an oncogene producing neoplastic transformation of the targeted mammary epithelial cells *in vivo* (Davies *et al*, 1996) and thereby enables immortalised mammary epithelial cell lines to be established in culture, as described in this report. Without overexpression of *neu* these cells remain normal and cannot be grown in culture (Jenkinson, 2001) without additional immortalising events. The tumours produced by the *neu* transgenic mice (Davies *et al*, 1996) or by a derived mammary epithelial cell line, when reintroduced into mice, are indolent and do not metastasise. These results are consistent with the correlation between the levels of Neu protein in the different cell lines and their growth rates in culture, but not with other properties measured including metastasising ability *in vivo*. Tissues of transgenic mice overexpressing S100A4, on the other hand, fail to undergo neoplastic transformation and that includes those of the mammary gland (Davies *et al*, 1995). However in *neu*-transformed neoplastic immortalised mammary cell lines, *in vitro* studies demonstrate that S100A4 levels correlate well with cell invasion and that S100A4 mediates, either directly or indirectly, this invasion through its ability to induce cell motility, at least in the transfected cells. It therefore seems probable that S100A4-driven cell motility mediates invasion *in vivo* and thereby metastasis *in vivo*, at least in this particular model system.
